# Safety and Feasibility of Outpatient High Dose Cytarabine for Acute Myeloid Leukemia in the Brazilian Amazon

**DOI:** 10.18502/ijhoscr.v14i3.3722

**Published:** 2020-07-01

**Authors:** Amanda Lopes Maia Rodrigues, Daniel Macêdo do Nascimento, Josy Marinho de Lima, Marcos Laércio Pontes Reis, Lucyana Barbosa Cardoso Leão, Murilo Chermont Azevedo, Samanta Ribeiro Muccini, Polyana Castanha da Silva, Thiago Xavier Carneiro

**Affiliations:** 1 School of Medical Sciences, Pará State University, Belém – PA, Brazil; 2 Division of Hematology-Oncology and Stem Cell Transplantation, Ophir Loyola Hospital, Belém – PA, Brazil; 3 School of Medical Sciences, Federal University of Pará, Belém – PA, Brazil.

**Keywords:** Acute myeloid leukemia, High drug dose, Chemotherapy, Outpatient care, Low-income population

## Abstract

**Background:** The attempt to manage patients with acute myeloid leukemia as outpatients has become increasingly common due to high hospitalization costs, low availability for beds and patient preference. Publications on the subject are scarce, especially in low-income regions and the safety in this population remains to be determined. The present study aims to assess the safety of consolidation with high-dose cytarabine in the outpatient setting.

**Materials and Methods:** We retrospectively analyzed 39 patients who underwent consolidation with high-dose cytarabine, between 2009 and 2018, at Ophir Loyola Hospital, in Belém, Brazil. Patients treated after 2015 were given high-dose cytarabine as outpatients due to the decision of medical staff.

**Results:** Twenty-seven patients received 76 cycles of cytarabine as outpatients; males were 48.14% of the total population, with a median age of approximately 45 years. The occurrence of delay between cycles was significantly lower among outpatients (48.14% vs. 83.33%, p = 0.04). There was no difference in relapse rates, transfusion requirements and non-relapse mortality between both groups. Hospitalization was required in 40.74% of patients during outpatient cycles and 18.51% of blood cultures were positive for pathogens. Non-relapse mortality was significantly higher among patients above 50 years old and treated on an outpatient basis (44.4% vs. 5.60%, p = 0.03).

**Conclusion:** High-dose cytarabine administration on an outpatient basis appears to be safe and effective in a low-income population at the Brazilian Amazon region, but toxicity seems to be increased for patients older than 50 years.

## Introduction

 Acute Myeloid Leukemia (AML) is an aggressive disease that requires intensive treatment, usually consisting of induction chemotherapy and consolidation with high-dose chemotherapy or stem cell transplantation ^1^. Multiple cycles of cytarabine at high doses (6000-18000 mg/m²/cycle) have been used as consolidation treatment of patients with acute myeloid leukemia, especially in patients younger than 60 years, with low risk AML ^2^^,^^3^^,^^4^.

Due to the potential risk of complications resulting from prolonged neutropenia, post-induction chemotherapy has traditionally been given on an inpatient basis to patients remaining hospitalized until hematologic recovery ^5^. In many countries, an average hospitalization period of 3 to 4 weeks for each cycle is adopted ^6^.

Prolonged hospitalization, however, increases treatment costs, leads to persistent exposure to hospital-acquired and often multidrug-resistant organisms and impacts on quality of life, increasing rates of depression and sleep disturbances^1^^,^^7^^,^^8^. Outpatient treatment can result in a reduction of hospital stay, shorter duration of febrile neutropenia treatment and fewer nosocomial infections^9^^,^^10^^,^^11^^,^^12^.

The viability, economic impact and safety of the outpatient regimen for high-dose cytarabine consolidation therapy has been previously assessed^1^^,^^13^^,^^14^^,^^15^^,^^16^^,^^17^^,^^18^^,^^19^, without significant differences in the incidence of complications ^5^. None of these reports, however, were performed in low income regions ^20^^,^^21^. 

The benefits of outpatient treatment are even more relevant in developing countries considering costs, access and hospital bed occupancy ^22^. It is important to assure the feasibility of the outpatient strategy in this setting. 

The present study aims to analyze the safety and feasibility of outpatient consolidation with high-dose cytarabine in a low-income population.

## MATERIALS AND METHODS

 This is a retrospective study carried out at Ophir Loyola Hospital (Belém, Brazil). This is the single public health tertiary hospital for treatment of hematologic malignancies in the state.

Data were obtained by chart review of 39 patients diagnosed with AML treated at this hospital from 2009 to 2018, aged ≥ 16 years, who underwent induction therapy, attained complete remission and received consolidation with high-dose cytarabine at a dose of 3g/m^2^, twice a day, on days 1, 3 and 5. For patients above 60 years of age, the dose was reduced to 1g/m^2^ in the same schedule. 

Patients treated between 2009 and July 2015 received cytarabine as inpatients. After this date, all patients were treated in an outpatient setting. The protocol shift was adopted by the hematology department, motivated by the high demand and limited bed availability. Registers before 2009 could not be accessed. 

Patients were clarified and oriented about their disease and care. Medical and transfusion support to outpatients occurred in a day hospital facility, with all patients being advised to return to the hospital in case of fever (> 38ºC) or change in clinical status. Oral antimicrobial prophylaxis consisted of acyclovir 800mg/day, fluconazole 300mg/day and ciprofloxacin 500mg/day. Transfusion requirements were determined by blood counts and clinical status.

The studied variables included sex, age, inpatient/outpatient consolidation, date of consolidation cycles, cycle delay, readmissions, complete remission, relapse and death within 30 days after cytarabine cycle (early death). Statistical analysis was performed using SPSS statistics® software and a p value of 0.05 was used as the cut-off for significance.

This research complies with the National Health Council's Research Guidelines Involving Human Beings (Res. CNS 466/12) and the precepts of the Declaration of Helsinki. Approval was obtained from the Research Ethics Committee of Ophir Loyola Hospital, CAAE 00675318.0.3001.550 and opinion 3.121.297, on January 24, 2019.

## Results

 Between 2009 and 2018, 12 (30.76%) patients received high-dose cytarabine on an inpatient basis and 27 (69.23%) on an outpatient basis. 58.33% (7/12) of inpatients and 48.14% (13/27) of outpatients were male. Patients receiving outpatient chemotherapy were older than hospitalized patients. Transfusion requirements were slightly higher among outpatients. (Table 1).

**Table 1:** Patient characteristics, consolidation cycles and transfusion requirements

	Outpatient N = 27	Inpatient N = 12	p
**Sex (%)** **Male**	48.14%	58.33%	0.59
**Age (years)** **Median (range)**	45.45 (18.66 - 66.21)	30.24 (16.52 – 51.38)	0.93
**Cytarabine cycles** **Median (range)**	3 (2 - 5)	3.5 (2 - 5)	-
**Red blood cell** **concentrates** **Mean (range)**	4.18 (0 - 14)	2.41 (0 - 12)	0.10
**Platelet** **concentrates** **Mean (range)**	12.37 (0 - 54)	10.41 (0 - 50)	0.13

Hospitalized patients received 42 cycles of high-dose cytarabine and outpatients received 76 cycles. The occurrence of delay (> 7 days) in at least one cycle was significantly higher among inpatients, with similar relapse rates (Table 2).

**Table 2:** Patients’ main outcomes (delay, relapse and early death)

	Outpatient N= 27	Inpatient N = 12	p
**Delay (%)**	48.14%	83.33%	0.04
**Relapse (%)**	40.74%	50%	0.42
**Early death (%)**	18.51%	16.66%	0.63

Therefore, 25% of outpatient cycles (19/76) required a short admission period after cytarabine administration. 18.51% (5/27) presented positive bloodstream culture. Agents identified were: *Escherichia coli* (60% of positive cultures), all resistant to quinolones and sensitive to 4th generation cephalosporins and carbapenems; *Pseudomonas aeruginosa* (20% of positive cultures), susceptible only to colistin; and *Staphylococcus epidermidis* (20% of positive cultures), sensitive to all tested antimicrobials. None of the patients with a positive blood culture died.

The early death rate was similar in both groups, all in remission. No patient had any significant treatment-associated neurotoxicity. However, in outpatients aged 50 years or older, a higher mortality was observed when compared to younger patients. Nonetheless, delays were significantly lower, and all deaths occurred in remission. Transfusion requirements and relapse rates were similar in both groups (Table 3).

**Table 3:** Main outcomes and transfusion requirements among outpatients aged above and below 50 years

	Age <50 (n = 18)	Age >50 (n = 9)	*p*
**Early death (%)**	5.60%	44.40%	0.03
**Relapse (%)**	50%	22.22%	0.16
**Delay (%)**	66.66%	11.11%	0.008
**Red blood cell** **concentrates** **Mean (range)**	3.55 (0 - 14)	5.44 (0 - 9)	0.44
**Platelet concentrates** **Mean (range)**	10.72 (0 - 44)	12.88 (0 - 38)	0.07

## Discussion

 In Brazil, to our knowledge, this is the first report of high-dose cytarabine consolidation on an outpatient basis. One of the major concerns about implementing this system is the occurrence of delays in administration of cycles, especially due to limitations in access or transportation of patients to the hospital, notably more pronounced in a large country such as Brazil. However, in the present study, significantly lower delays were found among outpatients. This shows that access is probably not a limitation to outpatient consolidation. Other studies also showed a similarity between inpatients and outpatients regarding periodicity ^19^.

In addition, the occurrence of delays or other complications during outpatient administration could impact the incidence of relapse among patients. In previous studies, however, significantly lower rates of relapse were reported among outpatients (32% vs 77%)^19^. There was no difference in relapse rates between groups in our population.

The expressive need for transfusion after chemotherapy is also a concern. In this study, outpatients received a few more transfusions but there were no significant differences compared to inpatients, as shown in previous reports^5^. This is probably due to a tendency to adopt early transfusions for outpatients, with less tolerance, while inpatients can be closely watched for longer. It should be noted that there were no cases of significant bleeding in either group.

Administration of outpatient, high-dose cytarabine could also be limited by the classically reported elevated rate of neurotoxicity associated with treatment (8-20%)^23^. In the present study, none of the subjects presented significant neurological signs, reinforcing the findings from previous studies which have already demonstrated lower rates in recent years (0.7%) ^23^.

The number of cycles requiring hospitalization was similar to that previously reported (28%), although the number of patients admitted was higher (20%)^19^. However, transference to the hospital system occurred immediately, reinforcing the safety of this regimen to ensure adequate care for patients’ demands. Nevertheless, considering an estimated 40% to 33% reduction of treatment costs when adopting the outpatient regimen^5^^,^^24^, these results suggest a significant economic impact. It’s also described a better quality of life among patients that experienced a reduction in the number of hospitalized days ^25^.

The prevalence of positive blood cultures was much lower than previously reported for patients in outpatient consolidation (59%)^26^, and was close to rates described for hospitalized patients (14%)^13^. Corroborating data from the literature, prophylaxis given to outpatients promotes a greater occurrence of resistance to quinolones, especially during the 2nd cycle of consolidation^27^. However, even with the isolation of a multidrug resistant pathogen, reduction of sepsis occurrence in patients treated in the outpatient setting ^10^ is reinforced, and there was an absence of deaths among patients with positive culture.

The early mortality rate was higher than internationally reported for newly diagnosed patients undergoing induction chemotherapy (4%)^28^. However, there was no significant difference between inpatients or outpatients, possibly reflecting the expected values ​​in the context of the country's population, according to previous surveys (mortality during consolidation of 21-24%) ^29^^,^^30^.

Finally, although there are studies showing the safety of outpatient consolidation in elderly patients^31^^,^^32^ using 2 cycles of cytarabine, in our hospital, mortality rates were higher among patients older than 50 years. This is possibly due to a higher prevalence of comorbidities or increased toxicity and infection rates during longer consolidation treatment, evidencing the need for differentiated support strategies and discouraging adoption of this regimen in this specific group of individuals. It is worth mentioning that this research has many limitations. For instance, it is a retrospective analysis based on medical records. In addition, we were unable to directly quantify the economic impact of outpatient treatment. Furthermore, this study represents the situation in a single tertiary hospital, and the results will need to be reproduced in other institutions. 

Therefore, high-dose cytarabine administration on an outpatient basis appears to be safe and effective in the context of developing regions, possibly due to significantly reducing costs related to treatment and improving patient’s quality of life. However, such a regimen should be used with caution in patients over 50 years due to the high risk of toxicity in this group.
